# Construction Notes

**Published:** 1893-04-29

**Authors:** 


### CONSTRUCTION NOTES.

Five hundred pounds has been received by the Committee of the Glasgow Samaritan Hospital for Women towards the capital account of the trustees of the Bellahouston Fund. It has been decided to erect a hospital to provide accommodation for from twenty-five to thirty beds, on a site adjoining the Victoria Baths.

The Manchester Southern and Maternity Hospital.—Mr. John Ely, F.R.I.B.A., of Manchester, has designed these new buildings, which are to be erected on a site in Oxford-road, and facing the Whitworth Park, granted free of cost, save a low chief rent, by the Council of Owens College. The cost is estimated at about £12,000. The administrative block will face Oxford Road, and will be built of red brick and terra-cotta.

In the annual report of the Committee of the Hounslow Hospital, it was stated that it is decided to build a new ward forthwith, to be called the "Heston" Ward, in compliance with injunctions from the late Mr. W. Taylor, who contributed £500 for the erection of the same. Two additional bedrooms for the medical staff are also to be added. The whole of the sanitary arrangements are to be altered and improved, and the ventilation of the wards will be rendered more satisfactory.

The proposed hospital for infectious diseases at Stroud is to be planned by Mr. Milnes, who estimates that the cost of the building will be £4,000, exclusive of the cost of site and of the furniture. The hospital, the construction of which is to be proceeded with forthwith, is to be erected of brick, stone, and slate, and will consist of four blocks, *i.e.*, one administrative and three diseases blocks, to accommodate thirty-six patients (each ward containing two wards of six beds each), of which one block is to be reserved for small-pox. A piece of land will be purchased immediately adjoining the site chosen for the hospital for the purpose of isolation of the same, which site is on the north-east corner of the land at the workhouse belonging to the Stroud Union.

